# A Novel P-III Metalloproteinase from *Bothrops barnetti* Venom Degrades Extracellular Matrix Proteins, Inhibits Platelet Aggregation, and Disrupts Endothelial Cell Adhesion via α5β1 Integrin Receptors to Arginine–Glycine–Aspartic Acid (RGD)-Containing Molecules

**DOI:** 10.3390/toxins16110486

**Published:** 2024-11-09

**Authors:** Pedro Henrique de Caires Schluga, Debora Larangote, Ana Maria de Melo, Guilherme Kamienski Lobermayer, Daniel Torrejón, Luciana Souza de Oliveira, Valeria Gonçalves Alvarenga, Dan Erick Vivas-Ruiz, Silvio Sanches Veiga, Eladio Flores Sanchez, Luiza Helena Gremski

**Affiliations:** 1 Laboratório de Matriz Extracelular e Biotecnologia de Venenos, Universidade Federal do Paraná, UFPR, Curitiba 81531-980, Brazil; schluga@ufpr.br (P.H.d.C.S.); farmaciana.21@gmail.com (A.M.d.M.); guilherme.lobermayer@ufpr.br (G.K.L.); veigass@ufpr.br (S.S.V.); 2 Laboratório de Toxinologia de Venenos Animais, Fundação Ezequiel Dias, FUNED, Belo Horizonte 30510-010, Brazil; deboralarangote03@gmail.com (D.L.); luciana.oliveira.funed@gmail.com (L.S.d.O.); valeria.alvarenga@funed.mg.gov.br (V.G.A.); eladio.flores@funed.mg.gov.br (E.F.S.); 3 Laboratorio de Biología Molecular, Facultad de Ciencias Biológicas, Universidad Nacional Mayor de San Marcos, Lima 15081, Peru; daniel.torrejon@unmsm.edu.pe (D.T.); dvivasr@unmsm.edu.pe (D.E.V.-R.)

**Keywords:** snake venom metalloproteinase, disintegrin, animal venoms, *Bothrops barnetti*

## Abstract

Viperid snake venoms are notably abundant in metalloproteinases (proteins) (SVMPs), which are primarily responsible for inducing hemorrhage and disrupting the hemostatic process and tissue integrity in envenomed victims. In this study, barnettlysin-III (Bar-III), a hemorrhagic P-III SVMP, was purified from the venom of the Peruvian snake *Bothrops barnetti*. Bar-III has a molecular mass of approximately 50 kDa and is a glycosylation-dependent functional metalloproteinase. Some biochemical properties of Bar-III, including the full amino acid sequence deduced from its cDNA, are reported. Its enzymatic activity is increased by Ca^2+^ ions and inhibited by an excess of Zn^2+^. Synthetic metalloproteinase inhibitors and EDTA also inhibit its proteolytic action. Bar-III degrades several plasma and ECM proteins, including fibrin(ogen), fibronectin, laminin, and nidogen. Platelets play a key role in hemostasis and thrombosis and in other biological process, such as inflammation and immunity, and platelet activation is driven by the platelet signaling receptors, glycoprotein (GP)Ib-IX-V, which binds vWF, and GPVI, which binds collagen. Moreover, Bar-III inhibits vWF- and convulxin-induced platelet aggregation in human washed platelets by cleaving the recombinant A1 domain of vWF and GPVI into a soluble ectodomain fraction of ~55 kDa (sGPVI). Bar-III does not reduce the viability of cultured endothelial cells; however, it interferes with the adhesion of these cells to fibronectin, vitronectin, and RGD peptides, as well as their migration profile. Bar-III binds specifically to the surface of these cells, and part of this interaction involves α5β1 integrin receptors. These results contribute to a better comprehension of the pathophysiology of snakebite accidents/incidents and could be used as a tool to explore novel and safer anti-venom therapeutics.

## 1. Introduction

Snakes of the *Bothrops* genus (Viperidae family) are found throughout tropical and non-tropical Latin America, inhabiting a wide range of environments from deserts to tropical areas and reaching mountainous regions. All *Bothrops* snakes are venomous, and this genus is responsible for most of the human morbidity and mortality from snake bites in South America compared to any other venomous snake [1,2,3]. The species *B. barnetti*, also known as Barnett’s lancehead [4], is an endemic venomous pit viper predominantly found in the coastal desert of northern Peru and southeast of Ecuador [5,6]. It has a clinical relevance due to accidents involving humans, particularly in rural areas [7].

Envenomation by snakes of the *Bothrops* genus is characterized initially by localized symptoms, such as edema, inflammation and tissue injury, hemorrhage, necrosis, and blister formation. In addition, serious systemic effects involving hemostatic disturbances, hemorrhage, and coagulopathy may develop, such as bleeding events, venom-induced consumption coagulopathy (VICC), and cardiovascular shock resulting from hypovolemia and vasodilation [3].

A proteomic study revealed that the venom of *B. barnetti* shares a profile similar to that of other snakes within the same genus, e.g., *B. atrox* and *B. pictus* [8]. SVMPs are the predominant toxin proteins across viper venoms. Some *Bothrops* species contain as much as 74% of SVMPs in their venoms [9,10]. Based on their structural organization, SVMPs are subdivided into three classes, P-I to P-III. P-I consists only of a catalytic (M) domain, and P-II and P-III contain a Dis-like and cysteine-rich (C) domain in addition to M and Dis-like domains. Moreover, P-III can be subdivided into subclasses based on its post-translational modifications, e.g., catalytic processing between the M and D domains (P-IIIb), dimerization (P-IIIc), and complex formation with C-type lectin-like proteins (P-IIId) [11,12], and a new P-IIIe has recently been reported [13]. In *B. barnetti*, P-III SVMPs constitute 51.2% of the total proteins of venom, while class P-I represents approximately 22% based on proteomic data [8]. As reported for other P-III SVMPs, the severity of hemorrhage resulting from accidents with this snake seems to be correlated with the structure, high activity, and abundance of class P-III SVMPs [8,14]. Studies on this class of SVMP have highlighted two main characteristics: they are all proteolytic, possessing a functional metalloprotease domain, and (the majority have) higher hemorrhagic activity, as well as variable and specific biological effects [11,15]. Several disintegrins from SVMPs have an RGD motif. However, P-III SVMPs contain an XXCD sequence, which has been indicated to be relevant for multiple biological functions and target selection [16,17].

Venom-induced hemorrhage pathogenesis involves the direct damage of blood vessels by SVMPs [18]. The mechanism of hemorrhage, along with other hemostatic effects triggered by SVMPs, has been a target of numerous studies [14,19,20,21,22,23,24]. The degradation of the basement membrane (BM) and other components of the extracellular matrix of capillaries (ECM) has been suggested as a key event in the occurrence of blood vessel damage during envenomation pathogenesis [18,20]. The hydrolysis of BM components by SVMPs can significantly impact the stability of the endothelium, leading to bleeding. However, the catalytic activity appears to be similar in both hemorrhagic and non-hemorrhagic SVMPs, suggesting that the hydrolysis of BM components is not the sole mechanism contributing to the vascular damage caused by hemorrhagic toxins [18,24]. SVMPs also cleave critical proteins involved in the blood coagulation cascade and platelet aggregation. This proteolysis activates or inactivates proteins involved in these processes, thereby disrupting blood coagulation and platelet aggregation [22]. Several P-III SVMPs have been identified for their ability to inhibit platelet aggregation [14,25,26,27,28,29,30,31]. It is known that initial hemostasis is governed by two key platelet-specific binding receptors: GPVI, which binds collagen and fibrin(ogen), and GPIb-IX-V, which binds vWF and other key ligands [32]. Both receptors GPVI and GPIb work together to drive platelet activation and adhesion events under various blood conditions in the vasculature [33]. Furthermore, these receptors are regulated by metalloproteolytic regulation, where metalloproteinases cleave the receptors and release an ectodomain fragment into the bloodstream. Thus, these SVMPs typically bind to and/or cleave vWF, as well as the main collagen receptor GPVI or α2β1 integrin.

All of these studies support the notion that SVMPs and their domains and complexes are implicated in binding with integrin receptors, ECM proteins, and membrane platelet receptors. These proteinases have the ability to activate or inactivate proteins through enzymatic or non-enzymatic mechanisms, as well as interfere with tissue integrity, blood coagulation, and platelet aggregation, thereby contributing to the venom’s toxicity.

Herein, barnettlysin-III (Bar-III), a hemorrhagic class P-III SVMP found in the venom of the Peruvian snake *Bothrops barnetti*, was purified and characterized. In this paper, the biochemical and structural features of this enzyme are described. The characteristics of Bar-III pertaining to its effects on the hemostatic system, as well as the interference of this toxin on important endothelial cell processes, are also reported, and the possible mechanisms underlying these events are explored.

## 2. Results

### 2.1. Purification of Barnettlysin-III

**Figure 1:**
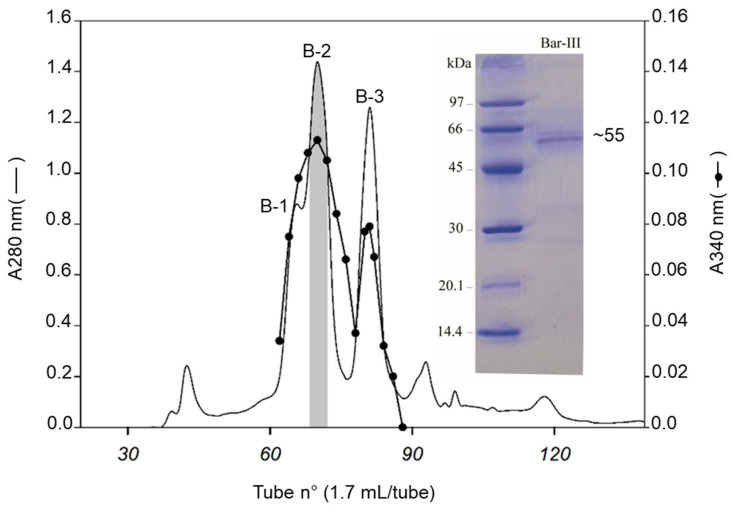
Purification of Bar-III. Active fraction from the second step (peaks D, E and F—Figure S2) was submitted to a Sephacryl S-200 column and equilibrated and eluted with HEPES buffer at pH 7.5, with 1 mM CaCl_2_ and 5 mM NaCl at a flow rate of 3.5 mL/h. The labels B-1, B-2 and B-3 denote the resulting peaks of this purification step. Inset indicates the purified Bar-III analyzed using SDS-PAGE (12% gel) under reduced conditions (5 µg). A band of ~55-kDa below the isolated proteinase may represent an autocatalytic fragment.

The SVMP Bar-III was isolated from *B. barnetti* venom by a three-step procedure, as previously described [34], with minor modifications. The purification process was monitored by determining the following for each fraction: hemorrhagic activity, proteolytic effect on DMC, fibrino(genol)ytic activity, and effect on platelet aggregation. Six peaks (P1, P2, P3, P4, P5, and P6) were obtained when 1150 mg of crude venom (1086 mg of protein) was submitted to a Sephacryl S-200 column (First step; Figure S1). The proteolysis of DMC was found in peaks P1 and P6. P1 (tubes 92–106) contained the high-molecular-mass proteins (~55 kDa), showing hemorrhage and DMC degradation, and the main hemorrhagic SVMP. This material was dialyzed with distilled water for 24 h and lyophilized. P6 (tubes 144–161) contained proteins of approx. 23-kDa showing proteolytic activity and traces of hemorrhage. A low Mr SVMP barnettlysin-I with a fibrinolytic effect but devoid of a hemorrhagic effect has been reported previously [35]. The low protein recovery (94 mg) may be explained by the presence of indissolvable materials and the degradation of proteins/toxins because *B. barnetti* venom contains a great diversity of active molecules represented mainly by metalloproteinases ~74.1% [8]. Total protein (94 mg) from the previous step was submitted to an ion-exchange column (DEAE Sepharose) with a pH 7.5 eluent, resulting in seven protein fractions (A, B, C, D, E, F, and G. Second step; Figure S2). The hemorrhage and proteolysis of DMC were observed in fractions D, E, and F. These fractions were pooled, lyophilized, and gel-filtered (45.4 mg) using a Sephacryl S-200 column. The three main peaks B-1, B-2, and B-3 (with an absorbance of 280 nm) were obtained (Figure 1). Both fractions possessed proteolytic activity upon DMC; however, hemorrhage was concentrated in peak B-2 (tubes 68–74). An aliquot of this fraction was applied to an RP-HPLC column, followed by analysis using MALDI-TOF and SDS-PAGE (12% gel). MALDI-TOF reveals a mass of 49,262 Da (Figure S3) and ~55 kDa by SDS-PAGE (Figure 1 inset). In this regard, it is known that carbohydrates are responsible for the microheterogeneity of glycoproteins, as observed using SDS-PAGE (Figure 1, inset). A 12.9 mg yield of active proteins resulted from 1150 mg of crude venom and presented a low recovery of 0.8% of total venom proteins.

### 2.2. Biochemical Properties of Bar-III

**Figure 2:**
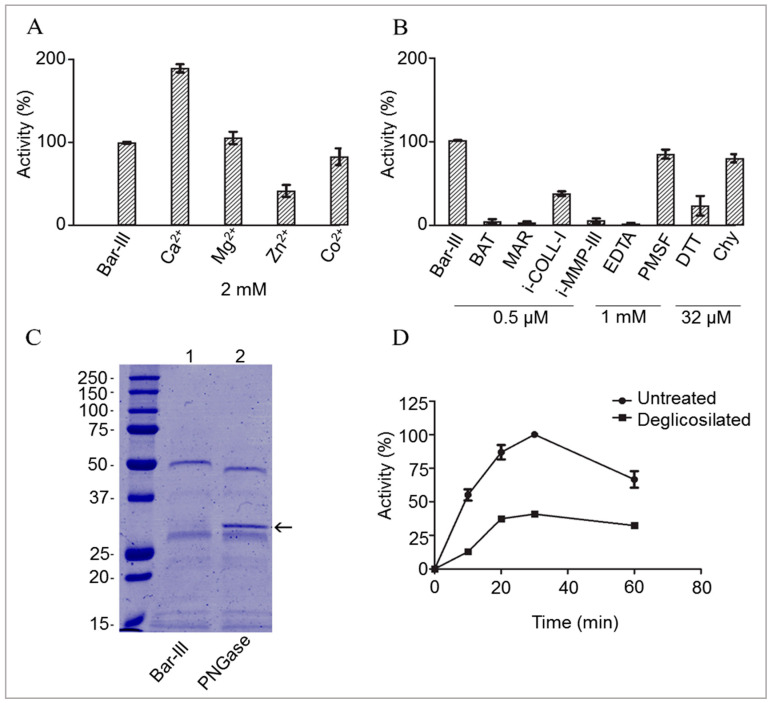
Some biochemical properties of Bar-III. (**A**) Effect of ions and (**B**) enzyme inhibitors on DMC proteolysis. Purified Bar-III (1.5 µg) was incubated with each compound in 250 μL of HEPES buffer (pH 7.5) for 15 min at 37 °C. Next, 250 µL of 2% DMC was added, and the activity was measured after a 30 min incubation at 37 °C. In addition, proteinase activity was challenged with various enzyme inhibitors: batimastat (BAT), marimastat (MAR), collagenase I inhibitor (i-Coll-I), MMP-III inhibitor (i-MMP-III), ethylenediamine tetra-acetic acid (EDTA), phenylmethylsulfonyl fluoride (PMSF), dithiothreitol (DTT), and chymostatin (Chy). (**C**) SDS-PAGE (12%) of native and deglycosylated enzymes (5 μg). Untreated Bar-III (control) or after incubation with PNGAse F: a band at approximately 34 kDa (right lane) is an excess of PNGAse F, and a band at approx. 30 kDa may correspond to P-II class SVMP. Marker proteins are indicated on the left. (**D**) Proteinase activity of Bar-III (5 µg) in the mixture was measured with DMC without (-●-) or after incubation with PNGAse F (-■-). Data are representative of SD (N = 3). Note that removal of N-deglycosylation drastically reduced enzyme activity.

The impact of ions and enzyme inhibitors on the activity of Bar-III on dialyzed micellar casein (DMC) was assessed, and the results are illustrated in Figure 2A,B. Zn^2+^ and Co^2+^ decreased the enzymatic activity of Bar-III, whereas Mg^2+^ had no effect on this trend. Conversely, Ca^2+^ ions augmented the proteolytic activity of Bar-III on DMC, indicating that Ca^2+^ is involved in the structural organization of SVMPs. However, the excess of Zn^2+^ significantly diminished Bar-III activity on DMC. To obtain complete deglycosylation of Bar-III, time-dependent assays were performed. Figure 2C shows the electrophoretic profile of Bar-III treated with Peptide-N-Glycosidase F (PNGase F), which cleaves N-linked oligosaccharides from glycoproteins. As observed, the band corresponding to deglycosylated Bar-III appears with a lower mass than the untreated enzyme, indicating that Bar-III is N-glycosylated. This post-translational modification is crucial for the proteolytic activity of Bar-III on DMC, as the removal of N-oligosaccharides after 30 min substantially reduced its binding and cleavage of substrates (Figure 2D).

### 2.3. Molecular Characterization of Bar-III

**Figure 3:**
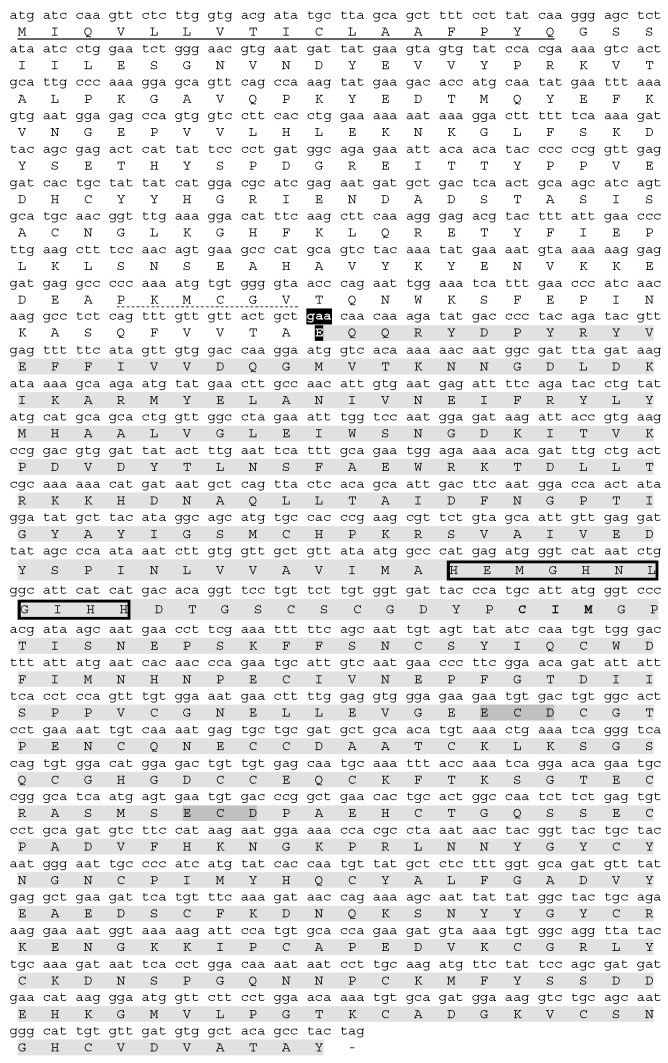
Full cDNA sequence of Bar-III precursor and its translated amino acid sequence. The signal peptide is underlined with a continuous line, and the consensus sequence in the propeptide (which controls the maturation of SVMPs) is underlined with a dotted line. The first residue of the mature protein Glu1 is highlighted in black. Mature protein sequence is highlighted in gray, and the typical zinc-chelating motif is marked with black box. The integrin-interacting motifs (ECDs) are highlighted in dark gray. The methionine-turn motif, CIM, is in bold. ORF termination by TAG codon is indicated by (-). The sequence of cDNA and the corresponding sequence of amino acids of Bar-III have been deposited in GenBank under accession number PQ268089.

A 1833-bp cDNA cloned in TOP10 *E. coli* encodes a protein of 610 amino acid residues containing a signal peptide (1–20), a pro-domain (21–189) with the activity-regulating cysteine-switch motif PKMCGVT, and a three-domain mature protein (Figure 3). The mature Bar-III (190–610) is a 47 kDa protein with a theoretical pI of 5.2, thirty-four conserved cysteine residues, and a potential N-glycosylation site (according to holoenzyme Bar-III numbering: Asn372). Like other P-III SVMPs, the Bar-III architecture exhibits metalloproteinase (M), disintegrin-like (D), and cysteine-rich (C) domains.

**Figure 4:**
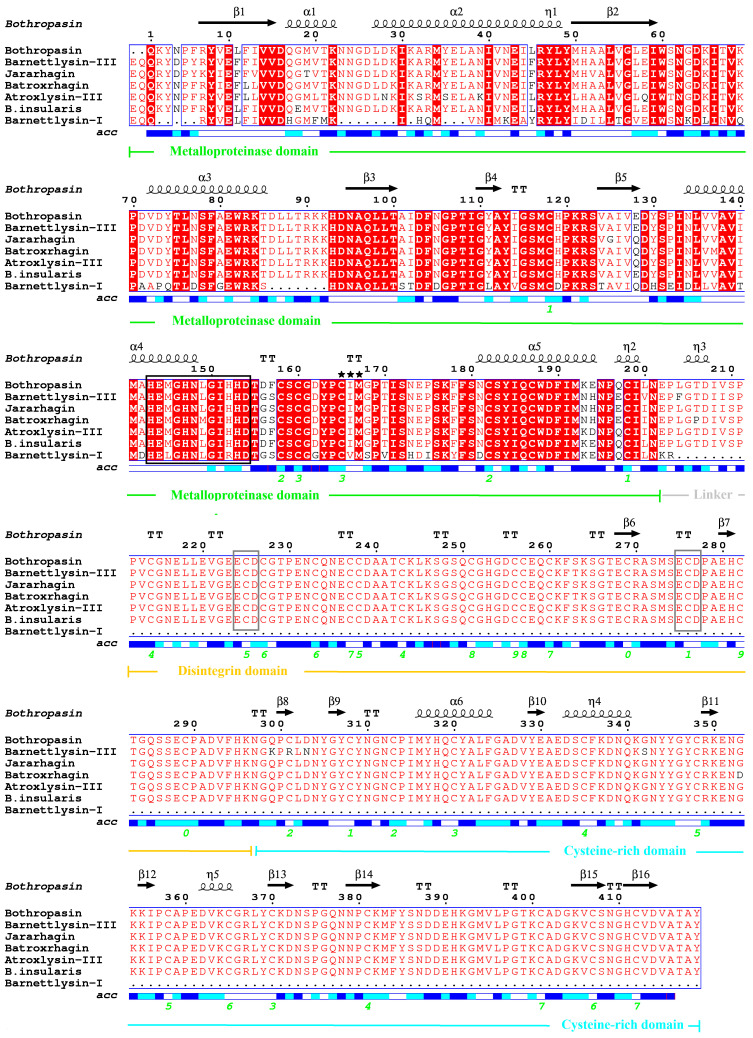
Multiple alignments of Bar III with other SVMPs from several *Bothrops* species. The red-colored residues represent the conserved regions throughout. The residues are numbered according to the alignment to Bothropasin (3DSL) and its secondary structural elements. The sequence of Bar-III is aligned with the following venom proteins: Atroxlysin III from Peruvian *B. atrox* (AQS99160), Bothropasin from *B. jararaca* (AAC61986), Batroxrhagin from Brazilian *B. atrox* (ALB00542), Jararhagin from *B. jararaca* (P30431), *B. insularis* P-III from *B. insularis* (AAM09693), and Bar-I, a P-I SVMP from the same *B. barnetti* venom (P86976). The zinc-binding motif (HEGNHLGIHHD) is in black box, and the invariant methionine 168 of the Met-turn region is shown with (★★★). Disintegrin-like (ECD) sequences are in gray boxes. Cys–Cys bonds are shown with green numbers. Surface accessibility is shown for modeled regions, with white, cyan, blue, and red representing buried, intermediated, accessible, and incomputable residues, respectively. The domains are indicated by lines and descriptions below the aligned sequences. Alignments and figures were generated by Clustal W 8.0. program and ESPript server, respectively.

The multiple sequence alignment with other homologous PIII SVMPs (Figure 4) shows a high homology of Bar-III with Bothropasin (95.5%) from *Bothrops jararaca*, Atroxlysin-III (93.1%) and Batroxrhagin (95.7%) from *Bothrops atrox*, BITM06A (95.3%) from *B. insularis*, and Jararhagin (96.4%) from *B. jararaca*. Interestingly, the limited variation among these SVMPs is concentrated in the M domain, which has 30 (14%) variable sites, compared to the D and C domains, which show 2 (2.3%) and 6 (5%) variable sites, respectively.

**Figure 5:**
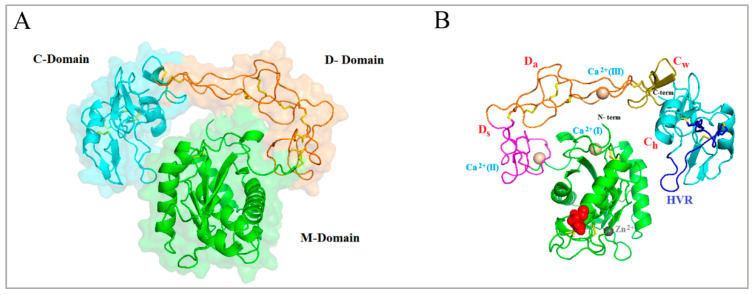
Theoretical 3D model of mature Bar-III. Bar-III architecture comprises three domains: metalloproteinase (M) (green), disintegrin (D) (orange), and cysteine-rich (C) (cyan). (**A**) Secondary structure elements (α-helices and β-sheets) are shown. Disulfide bonds are represented by yellow sticks. (**B**) The primary regions of Bar-III are depicted and include the M domain (green), and Ds (magenta), Da (orange), Cw (olive), and Ch (cyan) segments, along with the hypervariable region (HVR) (blue). Zinc and calcium ions bound to the structure are represented by gray and beige spheres, respectively. Sites for N-glycosylation are indicated by red spheres.

Structurally, the three-dimensional model of Bar-III was based on the structure of Bothropasin [36], showing 96% identity. The computational annotation of the 3D model of mature Bar-III (Figure 5) shows some conserved structural properties, such as a canonical Zn^2+^-binding motif (HEXHXXGXXH), a Met-turn motif, and a Ca^2+^-binding site in the M domain, two Ca^2+^-binding sites and an ECD motif in the D domain, and a hypervariable region (HVR) in the C domain. A linker sequence is located between the M and D domains, and another one remains between the D and C domains. The Ca^2+^-binding sites Ca^2+^(II) and Ca^2+^(III) are associated with the D shoulder (Ds) and D arm (Da) domains. The Ca^2+^-binding site (I), in turn, is situated opposite the zinc atom and in close proximity to the N-terminus of the M domain. The cysteine-rich domain is subdivided into C “wrist” (Cw) and C “hand” (Ch) (Figure 5B).

### 2.4. Proteolytic Activity of Bar-III upon Extracellular Matrix

**Figure 6:**
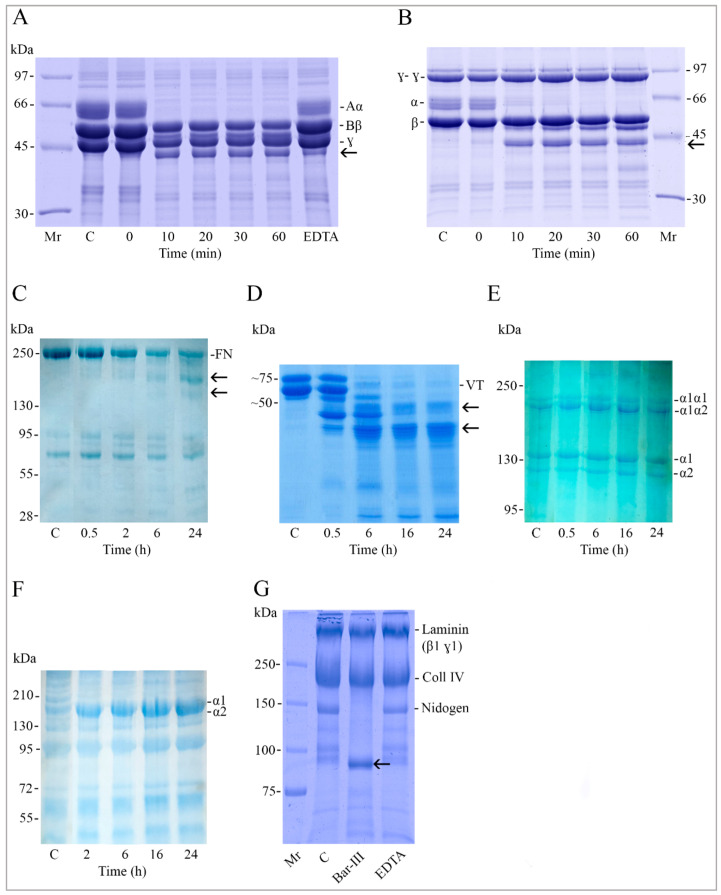
Analysis of fibrinogen, fibrin, fibronectin (FN), vitronectin (VN), type IV collagen, type I collagen, and Matrigel after treatment with Bar-III. Digestion reactions were performed at molar ratios of 1:100 (enzyme/substrate) of Fg (**A**), fibrin (**B**), and VN (**D**) and 1:50 for FN (**C**), coll-I (**E**), and coll-IV (**F**) for the indicated intervals at 37 °C. Aliquots of the incubation mixtures were analyzed by SDS-PAGE under reducing conditions on 10% gels to detect Fg and fibrin, 12.5% for VN, 7.5% for FN and coll-IV, and 5% for coll-I. The positions of the three polypeptide chains of Fg (Aα, Bβ, and γ) (**A**), along with the fibrin control γ-γ dimer and α- and β-chains (**B**), are indicated on the right and left, respectively. Typical coll-I chains (cross-linked α1α1- and α1α2-chain dimers, as well as monomeric α1 and α2) are indicated on the right (**E**). The position of the α1 and α2 domains of coll-IV are shown (**F**). Arrows at the right of (**A**–**D**) panels point to digestion products of ECM proteins. In panel H, 50 µg of Matrigel was incubated with 1.5 µg of Bar-III for 6 h at 37 °C. Reactions were terminated by addition of 10 mM of EDTA and analyzed using reduced SDS-PAGE (5–15% gradient gel). Control Matrigel (**C**), incubated with Bar-III, and EDTA-treated Bar-III are shown. The arrow at (**G**) panel points to a digestion product of Matrigel. Data are representative of three similar experiments.

Given that Bar-III is hemorrhagic, its activity on extracellular matrix (ECM) molecules and complexes was examined. The purified enzyme exhibits dose-dependent degradation of the α-chains of fibrinogen and fibrin under in vitro conditions. Fibrinogen was subjected to incubation with Bar-III for varying durations (0–60 min). Analysis via SDS-PAGE reveals that Bar-III functions as an α-fibrinogenase, degrading the α-chains within 10 min (Figure 6A). The α-chain primarily breaks down into a ~42 kDa fragment. The intensities of the Bβ- and γ-chain bands remain consistent over time, suggesting that these chains are not subjected to digestion. Similarly, the hydrolysis of fibrin by Bar-III results in α-chain degradation within the tested period (0–60 min) (Figure 6B).

In plasma, fibronectin (FN) exists as a dimer consisting of two structurally similar subunits (A and B), with sizes ranging from 210,000 to 250,000. FN interacts with multiple proteins, including fibrin, fibrinogen, and other FN molecules. Additionally, cross-linking by factor XIIIa is essential for incorporating FN into fibrin clots during coagulation, thereby enhancing structural stability [37]. Consequently, the impact of Bar-III on plasma FN was investigated. The patterns of human FN digestion are depicted in Figure 6C. The digestion products appear as two bands ranging from 130 to 250 kDa, with their intensities increasing over time and becoming more pronounced after 24 h.

Plasma vitronectin plays a role in processes following platelet stimulation, particularly in heparin binding, endothelial cell growth, and fibrinolysis [38]. Figure 6D illustrates two naturally occurring forms of vitronectin: a single-chain form (75 kDa) and a cleaved two-chain form (65 kDa). Enzymatic reaction with Bar-III led to the digestion of both bands after 6 h. The predominant degradation products exhibit apparent molecular weights below 50 kDa (Figure 6D).

To further elucidate the enzymatic function of Bar-III, its proteolytic activity on ECM molecules was investigated using type I and IV collagens. The SVMP exhibited no capacity to hydrolyze type I collagen. As depicted in Figure 6E, the α1α1- and α1α2-chains remained intact even after 16 h of incubation. In the case of type IV collagen, the molecule comprises two distinct polypeptides: the α1-chain (Mr~200 kDa) and the α2-chain (Mr~180 kDa). This basement membrane component remained undigested by Bar-III until 24 h (Figure 6F).

Given that laminin is a predominant component in all types of basement membranes, investigating its digestion by Bar-III is of interest. Thus, when the Matrigel complex was subjected to incubation with Bar-III, the digestion of nidogen became evident after 6 h (Figure 6G), while laminin remained intact, similar to its isolated form. Additionally, this basement membrane preparation shows the presence of type IV collagen (coll IV), which Bar-III did not degrade, in its purified form (Figure 6F).

### 2.5. Effects of Bar-III on Platelet Function

**Figure 7:**
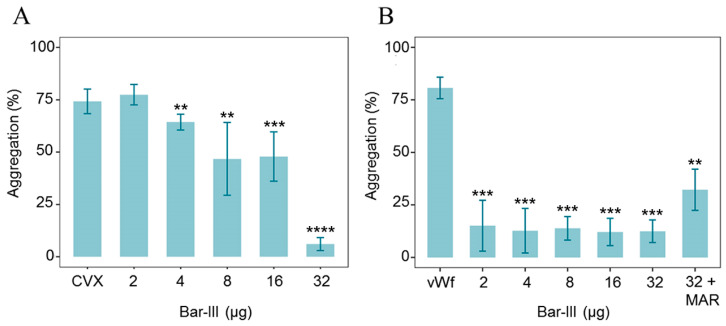
Effect of Bar-III on (**A**) convulxin (CVX)- and (**B**) von Willebrand factor (vWF)-induced platelet aggregation. Washed human platelets (WPs) (225 µL, 2.5 × 10^5^/µL) were pre-treated with various concentrations of Bar-III (ranging from 2 to 32 µg/mL for 3 min, with stirring at 600 rpm) at 37 °C, and then, different platelet agonists were introduced (CVX (6 µg/mL) and vWF (5 µg/mL) + marimastat (0.5 µg/mL)). Data shown are the mean ± SD of three independent experiments. ** *p* ≤ 0.005, *** *p* ≤ 0.001, **** *p* ≤ 0.0001.

The assessment of Bar-III activity in platelet aggregation triggered by two agonists was performed, and the results compiled in Figure 7 represent the average of at least three independent assays. Bar-III demonstrated a dose-dependent inhibition of platelet aggregation induced by convulxin (CVX), an agonist of GPVI and a physiological receptor of collagen unique to platelets (Figure 7A). Bar-III inhibited platelet aggregation induced by vWF at all tested concentrations by approximately 70%. This inhibition was partially reversed in the presence of marimastat (Figure 7B). The residual activity of Bar-III in the presence of marimastat suggests that this inhibition of platelet aggregation is likely not solely attributed to its proteolytic domain.

**Figure 8:**
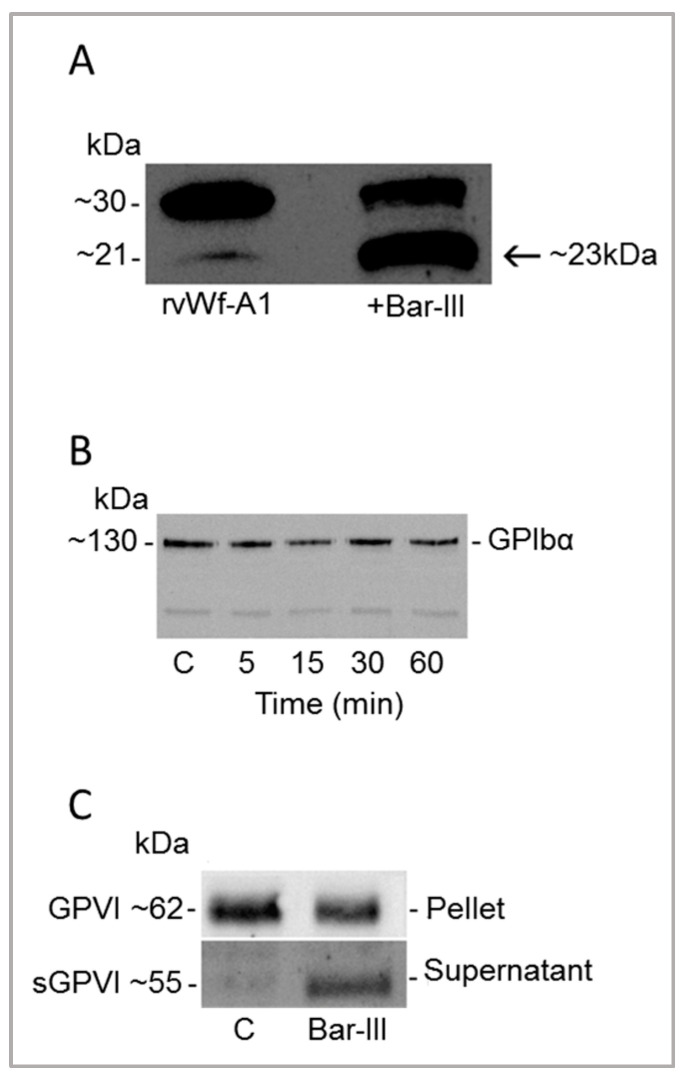
(**A**) Western blot analysis of Bar-III on recombinant vWF-A1 domain (rvWF-A1). vWF-A1 (5 µg) was treated with Bar-III (4 µg) for 60 min at 37 °C, as described in methodology section. The proteolysis of vWF-A1 (indicated by an arrow) was analyzed using SDS-PAGE (7–15% gradient gel) and blotted with mice anti-vWF IgG. (**B**) Bar-III does not cleave the vWF receptor partner GPIbα on platelets. Washed platelets were incubated with Bar-III (4 µg) at 37 °C at the indicated intervals, and the reactions were terminated by addition of SDS loading sample buffer. The platelet lysate was probed in WB with anti-CD42/GPIb. Note the intact ~130-kDa GPIbα expression on platelets. (**C**) Platelet pellets or supernatants of WPs treated with Bar-III (4 µg) for 60 min at 37 °C. The positions of GPVI (~62-kDa) and the soluble GPVI fragment (~55-kDa) are indicated. C, platelet control. These results are representative of at least three similar experiments for each item.

The activity of Bar-III on the A1 domain of recombinant vWF was assessed, along with its effects on the platelet receptors GPIb (a vWF receptor) and GPVI (a collagen receptor). Figure 8A shows that Bar-III cleaved the A1 domain of recombinant vWF, yielding a product of approximately 23 kDa. Conversely, in the kinetic assay with washed platelets shown in Figure 8B, the enzyme did not degrade the vWF receptor (GPIb) (~130 kDa). The same assay was conducted to investigate Bar-III’s activity on the collagen receptor (GPVI) (~62 kDa), and in Figure 8C, it is observed that the enzyme induced shedding of the ectodomain (~55 kDa) of this platelet receptor.

### 2.6. Viability Assays on Endothelial Cells

**Figure 9:**
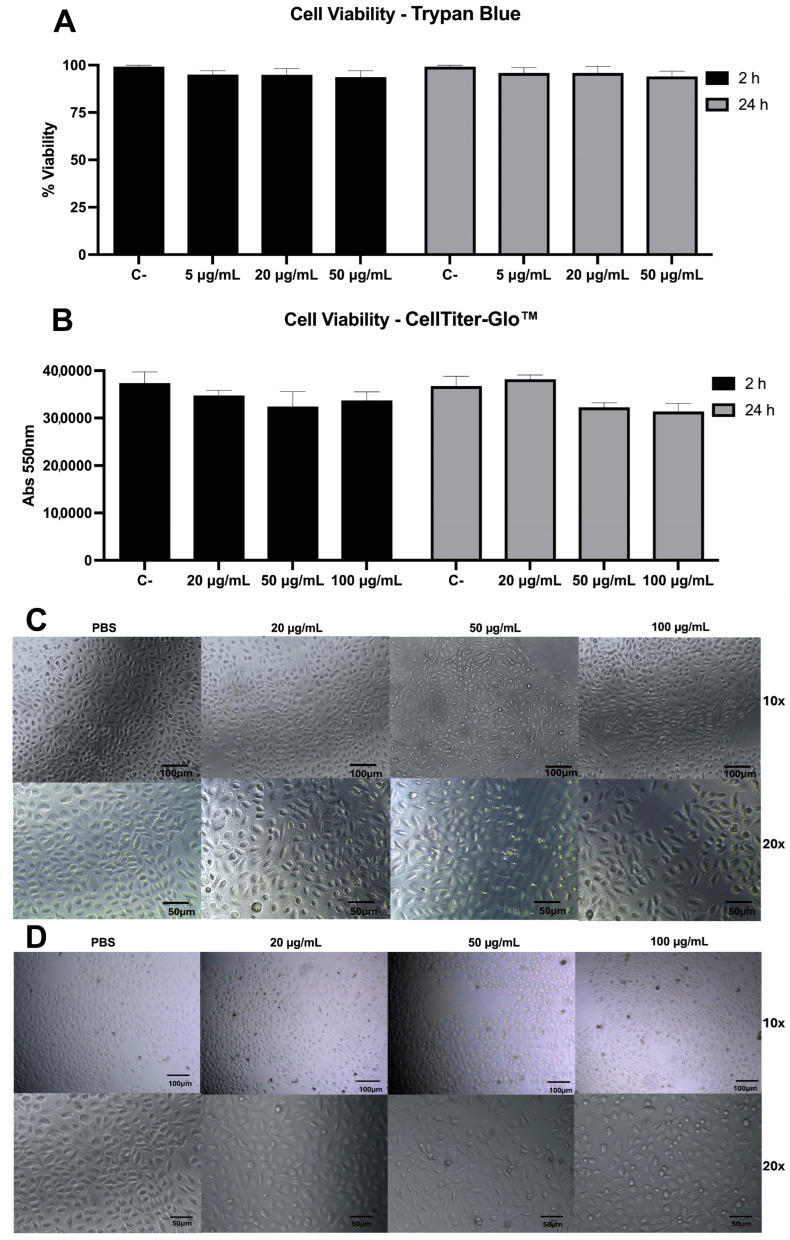
RAEC viability and morphology after exposure to Bar-III. (**A**) Cell viability accessed using trypan blue method. The toxin was incubated with adhered cells (5 μg/mL, 20 μg/mL, and 50 μg/mL) for 2 and 24 h and subjected to a cell viability test using the trypan blue method. Control used PBS instead of venom or Bar-III. No statistically significant difference was found between controls and treated groups (**B**) Cell viability was accessed using ATP quantification method. The toxin was incubated with adhered cells (20 μg/mL, 50 μg/mL, and 100 μg/mL) and subjected to a cell viability test using CellTiter-Glo Kit^®^. Control used PBS instead of Bar-III. No statistically significant difference was found between controls and treated groups. (**C**,**D**) Cells were incubated with increasing amounts of Bar-III (20 μg/mL, 50 μg/mL, and 100 μg/mL) for 2 h (**C**) or 24 h (**D**). Images of cells were acquired by Microscope Leica MPS30 (Leica Microscopy System Ltd., Heerbrugg, Switzerland).

The first cell viability test was carried out using the trypan blue method, which assesses cell membrane integrity. The assays were performed with Rabbit Aorta Endothelial Cells (RAECs) exposed to different amounts of Bar-III for different intervals. As observed in Figure 9A,B, the viability of cells did not decrease regardless of incubation time and concentration of Bar-III. The cell morphology (Figure 9A,B) corroborates the quantitative results, as no significant changes were observed after 2 or 24 h, even at high concentrations (100 µg/mL). There was a change in viability only when the cells were exposed to the *B. barnetti* venom, after 2 h and 24 h. To double-check the action of Bar-III on RAEC viability, an ATP quantification assay by luminescence was performed (CellTiter-Glo™ Kit PROMEGA, USA), as the ATP level is directly proportional to the number of viable cells in the sample. The results show that Bar-III did not affect RAEC viability, confirming the response observed in the trypan blue assay (Figure 9B). The cells treated with the venom of *B. barnetti* showed decreased viability after 24 h at the amounts of 50 μg/mL and 100 μg/mL.

### 2.7. Interference of Bar-III in Endothelial Cell Processes

**Figure 10:**
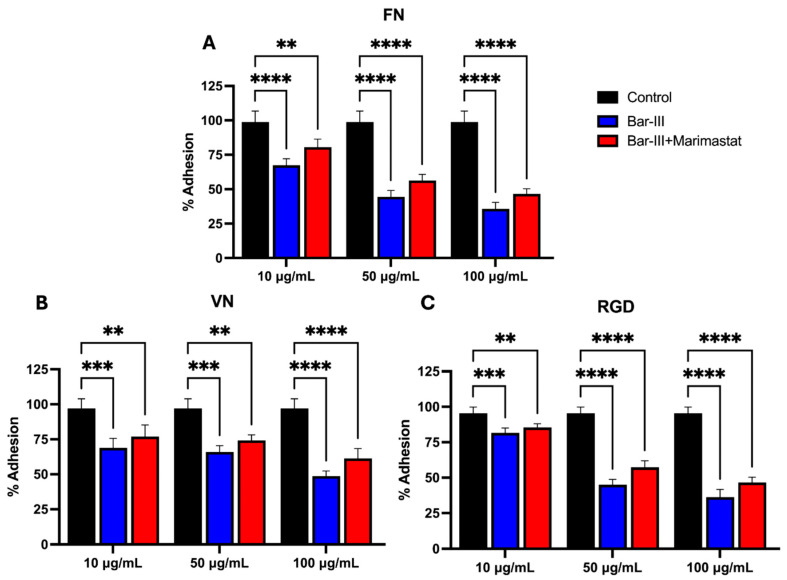
Cell adhesion of RAECs exposed to Bar-III on different extracellular matrix (ECM) molecules. Panel (**A**) shows cell adhesion to fibronectin (FN), panel (**B**) to vitronectin, and panel (**C**) to RGD peptide. Cells were incubated with Bar-III toxin at concentrations of 10, 50, and 100 μg/mL. The control condition used PBS instead of venom or Bar-III. Statistical significance was defined as follows: ** *p* < 0.01; *** *p* < 0.001; **** *p* < 0.0001.

To verify if Bar-III can interfere with endothelial cell adhesion to fibronectin, vitronectin, and RGD peptides, cells were incubated with Bar-III, and then, their adhesion to these molecules was assessed. Alternatively, cells were exposed to Bar-III toxin previously incubated with marimastat, which inhibits proteinase activity, as shown in Figure 10. The results show that both Bar-III and Bar-III inhibited by marimastat were able to reduce the adhesion of endothelial cells to all the substrates assayed (Figure 10). The fact that Bar-III devoid of proteolytic activity also hinders the adhesion of cells shows that such interference does not result from the proteolysis of proteins/peptides from the cell surface. 

**Figure 11:**
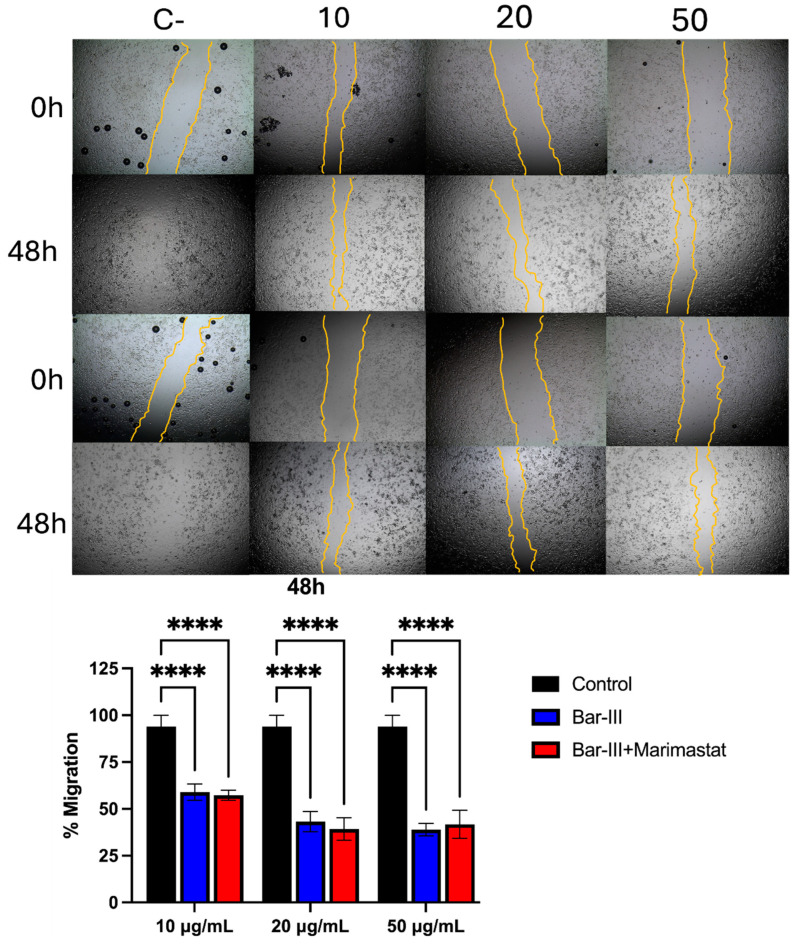
Assessment of cell migration by the scratch method in the presence of Bar-III in different amounts. The cell-free area was photographed at 0, 24, and 48 h. Analyses were carried out using Image J software v.1.8.0. **** *p* < 0.0001.

In addition, the results presented in Figure 11 show that the migratory profile of RAECs was impaired by Bar-III and Bar-III incubated with marimastat. This reduction in the migratory capacity is concentration- and time-dependent. Overall, these results demonstrate that Bar-III can interfere with the essential cell processes of cell adhesion, ECM, and cell migration and that such interference is not due to the catalytic activity of the metalloproteinase domain.

### 2.8. Interaction of Bar-III with Endothelial Cells via α5β1 Integrins

Additionally, polyclonal anti-Bar-III antibodies were produced in rabbits in order to use them as bio tools for other tests. Western blotting assays using Bar-III and *B. barnetti* venom demonstrate that these antibodies are suitable for detecting Bar-III (Figure S4—Supplementary Materials).

**Figure 12:**
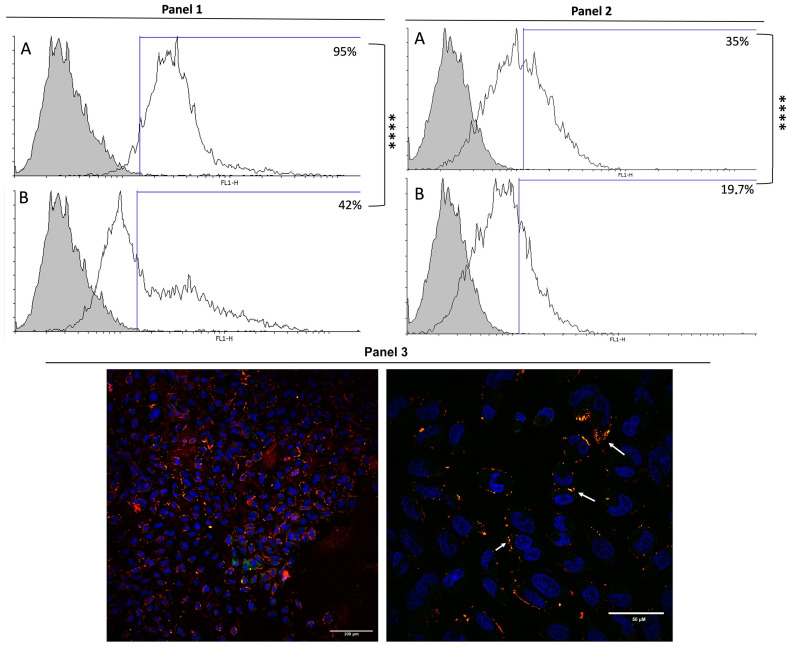
Bar-III interaction with RAEC surface and overlap of binding sites with α5 integrins. (Panel 1) RAECs were marked with polyclonal anti-Bar-III antibody and then incubated with anti-rabbit IgG Alexa 488-conjugated antibody. (**A**) Gray histogram indicates basal fluorescence (control) with secondary antibody; white histogram represents cells treated with Bar-III and marked with antibody, in which 95% of the cells were considered positive. (**B**) Gray histogram indicates basal fluorescence control with secondary antibody; white histogram is an antigen competition assay (pre-incubation of anti-Bar-III antibody with Bar-III solution incubated with RAECs exposed to Bar-III, showing 42% of marked cells, as shown in graph B (significance of **** *p* <0.0001). (Panel 2) Binding of anti-α5 antibodies to RAECs. RAECs were incubated with anti-α5 monoclonal antibody, which was recognized by mouse anti-IgG antibody conjugated with Alexa 488. (**A**) Gray histogram indicates basal fluorescence control with secondary antibody; white histogram represents cells marked with anti-α5 antibody; 35.1% of cells were positive. (**B**) Gray histogram indicates basal fluorescence control with secondary antibody; white histogram represents RAECs exposed to Bar-III, followed by incubation with anti-α5 antibody recognized by secondary antibody against IgG of mouse, showing 19.7% positive cells, as shown in graph C (significance of *** *p* <0.001). (Panel 3) Labeling of RAECs (nucleus stained with DAPI, Bar-III stained with Alexa 594, α-5 integrin Alexa-488), Arrows indicate regions of colocalization between Bar-III and integrins. 20× and 40× magnification.

The binding capacity of Bar-III on the surface of cultured endothelial cells was evaluated using the method of flow cytometric quantification of fluorescence. Briefly, cells were incubated for 30 min with Bar-III at subcytotoxic amounts, which were then recognized by an anti-Bar-III antibody. In this case, 95% of the cells were marked (Figure 12). The antigen competition assay demonstrates that Bar-III effectively inhibits the binding of antibodies to cells, as the measured fluorescence reduced to 42% (Figure 12). This result indicates that Bar-III acts as a ‘planted antigen’, interacting directly with the surface of endothelial cells in vitro.

To evaluate if Bar-III binds to α5 integrins on the surface of endothelial cells, cells were exposed to monoclonal antibodies that recognize α5 integrins (Figure 12). About 35% of the cells were positive for α5 integrin fluorescence, whereas only 19.7% of the test cells previously exposed to Bar-III showed fluorescence. This significant decrease in the fluorescent labeling of α5 integrins when cells were previously exposed to Bar-III indicates that some binding sites of anti-α5 integrin antibodies were unavailable due to their interaction with Bar-III.

In addition, experiments via confocal microscopy aimed to evaluate if Bar-III that binds to the RAEC surface co-localizes with α5 integrin receptors in cells. For this, cells were treated with Bar-III and then labeled to both Bar-III and α5 integrins. The results show a partial overlap of the two signals, indicating that part of the toxin binding to endothelial cells is via interaction with α5β1 integrins in cells (Figure 12).

## 3. Discussion

As reported by [8], the proteomic characterization of *B. barnetti* venom evidences the presence of at least two PIII SVMPs of approx. 50-kDa. In the present study, MS/MS analysis of a fragment (XTVKPDVDYTXNSFAEWR) obtained after the tryptic digestion of purified Bar-III reveals that this sequence fits perfectly within a Bar-III sequence (theoretical Mr of 52 kDa). The findings further reveal that Bar-III is a glycosylation-dependent functional metalloproteinase. This can be ascribed to the presence of a potential glycosylation site in Asn372. Clearly, SVMPs with larger molecular masses and high percentages of linked sugars are usually more hemorrhagic [39]. It is hypothesized that carbohydrates contribute to stabilizing the protein structure and modulating substrate binding and biological/cleavage activities. This assumption arises from the proximity of the N-linked glycoconjugate located at Asn181 to the “Met-turn” (Cys165, Ile166, Met167—CIM). The intrinsic flexibility of this region has been suggested as one of the critical factors contributing to SVMP-induced hemorrhage [40]. The observed reduction in proteolytic activity of deglycosylated Bar-III on dimethylcasein (DMC) compared to native Bar-III underscores the significance of the post-translational modification in this enzyme.

The three-dimensional model of Bar-III was derived from Bothropasin’s structure [36], exhibiting a 96% identity match. Calcium ions stabilize the structure of Bar-III, as proposed for other SVMPs/ADAMs and MMPs with collagenase activity [11,15]. These proteases have important functions in several inflammation and immune processes, as well as in ECM remodeling [11,39]. The alignment of the Bar-III sequence with other SVMPs highlights the conservation of both the number and positions of cysteine residues, which are highly conserved across the structures of SVMPs and ADAMs [41,42,43,44].

Additionally, the disintegrin domain is connected to the cysteine-rich domain via disulfide bonds. Finally, the hypervariable region (HVR) represents the segment with the highest amino acid sequence variability among the SVMPs. Various P-III SVMPs possess unique HVR sequences, leading to distinct surface characteristics. Therefore, the presence of this region, involved in protein–protein interactions, is recognized as one of the factors contributing to the various biological activities exhibited by this class of SVMPs [41].

The presence of the Met-turn motif provides a hydrophobic base for the binding of zinc. This feature aligns with the findings of enzymatic activity loss in Bar-III following previous treatment with EDTA. These characteristics are consistent across all members of the Metzincin superfamily. Moreover, similar to other P-III SVMPs, the MDC domains of Bar-III adopt a C-shaped configuration, with the distal portion positioned near the zinc ion in the catalytic site [45]. The classification of Bar-III as a class P-III SVMP is supported by the observed effects of Ca^2+^, Mg^2+^, EDTA, and metalloproteinase inhibitors (such as batimastat and marimastat) on its proteolytic activity [29,46,47,48]. The inhibition of Bar-III’s proteolytic activity by excess Zn^2+^ can be explained by the hypothesis that Bar-III likely possesses additional binding sites for Zn^2+^ with varying binding affinities, as suggested for Atr-III [14] and acurhagin [48]. The occupation of these sites with a high concentration of ions may induce a conformational change, leading to the subsequent loss of enzymatic function.

One notable aspect of the activities attributed to class P-III SVMPs is their capacity to induce severe hemorrhagic conditions by disrupting the hemostasis process or interfering with the interactions between cellular receptors and their ligands [49]. Plasma and extracellular matrix (ECM) protein degradation assays reveal Bar-III’s ability to selectively degrade the Aα-chain and partially the Bβ-chain of fibrinogen, along with the α-chain of fibrin. Furthermore, they demonstrate the degradation of components of Matrigel^®^, such as laminin β1ɣ1, collagen IV, and nidogen. Bar-III partially degrades fibronectin, which is involved in cell migration and adhesion, as well as playing a fundamental role in wound healing and hemostasis [37]. It also degrades vitronectin, which is important in platelet adhesion and aggregation mechanisms [50,51]. Numerous studies have highlighted the remarkable ability of SVMPs to degrade proteins involved in blood coagulation, such as fibrin, fibrinogen, and vWF [14,25,52], as well as basement membrane components and plasma proteins [15]. Such activities have also been observed in vivo through analyses of exudates from tissues treated with these toxins [19,53]. Moreover, fibrinolytic metalloproteases can inhibit interactions between platelet surface receptors and collagen or vWF [54,55,56].

Platelet adhesion to collagen triggers the initiation of the clotting response, while the activation of platelets induced by collagen further accelerates this process by releasing granule contents and activating platelet integrins [57]. GPVI is one of the direct collagen receptors on platelets, and its deficiency results in the loss of platelet response to collagen in humans and mice [58,59,60]. Downregulation of GPVI can be induced by cleavage of its ectodomain, a process dependent on ADAM10 and triggered by the activation of the coagulation cascade or exposure to shear stress. Bar-III induced the shedding of GPVI, resulting in the release of a soluble fragment of the ectodomain. The shedding of GPVI by Bar-III may additionally contribute to its inhibitory effect on convulxin-induced platelet aggregation, consistent with findings involving the homologous P-III class SVMPs Atr-III (*Bothrops atrox*) and AAVI (*Agkistrodon acutus*) [14,61].

Bar-III also inhibited platelet aggregation when von Willebrand factor (vWF) was used as an agonist. vWF serves two primary physiological roles in preventing bleeding: transporting coagulation Factor VIII and facilitating the formation of platelet aggregates [55]. Under normal circumstances, vWF circulates independently without interacting with platelets. However, upon vascular injury, when basement membrane components, like collagen, are exposed, vWF increases its affinity for circulating platelets [62,63]. Bar-III also demonstrates the ability to cleave the recombinant A1 domain of vWF, which partially elucidates its inhibitory impact on platelet aggregation induced by vWF. The association of the vWF A1 domain with GPIb has been documented in the literature [64]. Based on the recent literature, the key platelet receptors GPIb and GPVI play critical roles in thrombus formation and stabilization; thus, the use of non-hemorrhagic P-I SVMPs, e.g., mutalysin-II, which efficiently dissolved thrombus in in vivo models without affect hemostasis, may represent an interesting model to develop new anti-thrombotic drugs [56].

Endothelial cell damage correlates with capillary hemorrhage in vivo. Ultrastructural examinations of endothelial cells from tissues treated with SVMPs reveal significant pathological alterations in these cells [65]. Herein, in vitro assays demonstrate that Bar-III did not reduce the viability of endothelial cells but induced a slight rounding of these cells. A previous study demonstrated that endothelial cells in culture, while being resistant to changes in viability when exposed to various SVMPs, can undergo morphological alterations and subsequently detach from their substrate [66]. This effect is potentially attributed to the cleavage of ECM proteins rather than a direct action on the cells themselves. The ECM of these cells has been previously reported to contain a substantial amount of fibronectin, which is a target of Bar-III [67]. This detachment from the substrate may ultimately lead to apoptosis, as observed in endothelial cells treated with P-III SVMPs, such as jararhagin, HV-1 from *Trimeresurus flavoviridis*, and Atrase A from *Naja atra* venoms [68,69,70]. Some studies have demonstrated that SVMPs can break down cell adhesion and intercellular connections via proteolytic or disintegrin-like domains, resulting in apoptosis [71,72]. Future research could explore whether apoptosis-related mechanisms are activated upon exposure to Bar-III.

The ECD motif of SVMPs is often involved in interactions with integrins or other cell surface receptors. This motif appears densely packed near the C domain, which may hinder its interaction with integrins [73,74]. However, some studies report the interaction of PIII SVMPs with integrins, leading to the subsequent disruption of associated events, including adhesion, proliferation, and migration [75,76]. Therefore, tests were performed to assess the impact of Bar-III on the adhesion process of endothelial cells in a culture with various RGD-containing ECM molecules (FN and VN) and RGD peptides. The results show interference in the adhesion profile of cells to these molecules, which appears to be independent of Bar-III’s proteolytic activity. The migration profile of these cells was likewise reduced in the presence of Bar-III, an outcome also independent of enzymatic activity. Immunostaining reveals a specific interaction of this toxin with the surface of endothelial cells. It indicates that a portion of this toxin binds to α5β1 integrin receptors on the cell surface, which are recognized as fibronectin and RGD ligands [77]. Together, these results indicate that the impairment of adhesion to fibronectin, vitronectin, and RGD peptides, as well as the decrease in migratory capacity, may be partially attributed to the binding of the toxin to α5β1 integrin sites. These findings suggest that the toxin’s effects on cells are complex. This complexity likely contributes to the variability in cellular responses observed among SVMPs and with different cells.

Vitronectin, an abundant plasma protein, binds to activated platelets, facilitating platelet adhesion and aggregation. Furthermore, thrombi formed due to vascular injury exhibit instability when vitronectin is absent. This is because vitronectin binds and stabilizes plasminogen activator inhibitor-1 (PAI-1), potentially protecting fibrin from degradation and functioning as a thrombus stabilizer [78]. Disruption of the binding between endothelial cells and fibronectin, typically mediated by integrins, can interfere with processes, including proliferation, migration, and differentiation, ultimately rendering the cells nonviable [79]. Since not all α5β1 integrin sites were occupied by Bar-III in the flow cytometry analysis, even with an excess of this toxin in the solution, it is possible to infer that the densely packed ECD motif may hinder the interaction of Bar-III with integrins. A study compared the action profiles of jararhagin (PIII SVMP) with the sole catalytic domain of this toxin, known as jari [80]. The evidence indicates that jari exhibited greater activity against B16F10 cells in terms of cytotoxicity and reducing proliferation compared to the full molecule. This report supports our findings, indicating that the obstruction of access by the ECD motif to its cellular ligands in both jararhagin and Bar-III makes interaction with cells difficult. Nevertheless, upon interacting with these receptors present on endothelial cells, Bar-III disrupts their interaction with ECM molecules, which is essential to processes such as hemostasis, healing, and tissue cohesion maintenance, thereby hindering these events. These observations are significant when considering in vivo conditions. For instance, even when its catalytic activity is blocked by natural tissue inhibitors, Bar-III may still disrupt crucial tissue functions. Platelet aggregation assays with Bar-III and marimastat show its effect on coagulation, and its impact on tissue repair is evident in studies with cultured endothelial cells. However, it is important to note that Bar-III’s effect on the hemostatic process is not only due to its disintegrin motif but also its ability to degrade blood coagulation-related substrates and adhesion receptor proteins on the ECM and platelets. Furthermore, molecules that impair the interaction between ECM ligands and cells have shown potential in generating drugs with activity in inhibiting platelet aggregation by blocking RGD-dependent integrin sites [81,82,83,84]. Additionally, other properties of such molecules show promise in the study and development of new drugs, including their ability to inhibit tumor metastasis [85] and angiogenesis in tumors [86].

## 4. Conclusions

Taken together, the results indicate that the P-III SVMP Bar-III plays an important role in the hemorrhage and tissue disruption observed after a bite by *B. barnetti*, as it interferes with the integrality of ECM molecules and coagulation factors involved in such processes and reduces the interaction of endothelial cells with important integrin receptors. The study of this protein contributes to the understanding of the clinical manifestations of snakebite accidents. Additionally, it represents a potential avenue for exploring new therapeutic options, such as the development of anti-thrombotic medications or even novel anti-angiogenic and anti-metastatic drugs for cancer treatment.

## 5. Materials and Methods

### 5.1. Venom

Venom of the *B. barnetti* (Barnett’s lancehead) was obtained from specimens captured in the arid coastal region of Talara, Dept. of Tumbes, Pacific northern Peru, and kept in captivity at the Serpentarium Oswaldo Meneses, Museo de Historia Natural, Universidad Nacional Mayor de San Marcos (UNMSM), Lima-Peru. The handling of snakes to extract venom complied with the current legislation established by the National Council for the Control of Animal Experimentation (CONCEA) and approved by CEUA/FUNED (protocol No. 015/2019).

### 5.2. Chemicals and Reagents

Human fibrinogen (Fg) (essentially plasminogen-free), fibronectin (FN), and dimethylcasein (DMC) were purchased from Sigma Chemical (St. Louis, MO, USA). N-glycosidase F (PNGAse F, P0704S) was acquired from New England Biolabs (Ipswich, MA, USA). Type I collagen (5368) was acquired from Helena Laboratories (Beaumont, TX, USA). Prostaglandin E1 (P5515) and von Willebrand factor (vWF, 681,300) were purchased from Merck (Darmstadt, Germany). The GPVI polyclonal antibody (AF3627) of human platelets and CD42b/GPIbα (antihuman) polyclonal antibody (AF4067) were acquired from R&D Systems (Minneapolis, MN, USA). All other chemicals were of reagent grade.

### 5.3. Barnettlysin-III Purification

The metalloproteinase (SVMP) barnettlysin-III (Bar-III) was purified from Peruvian pit viper *B. barnetti* venom using gel filtration using a Sephacryl S-200 combined with ion-exchange chromatography on a DEAE Sepharose CL 6B, according to methods previously described [34], with minor modifications. For the first step, 1.086 mg protein in 8 mL of 50 mM ammonium acetate buffer (pH 7.3) with 0.3 M NaCl was applied to two series (2.5 × 100 cm) columns packed with Sephacryl S-200 and equilibrated and eluted with the same buffer. The flow rate was kept at 7 mL/h, and 5.8 mL fractions were collected at 4 °C. Active fractions were pooled and concentrated using a Centricon microcentrifugal concentrator (Amicon, Inc., Danvers, MA, USA). For all chromatography protocols, the protein concentration was assayed by determining the absorbance at 280 nm. Proteolytic activity was tested with DMC as the substrate, and SDS-PAGE on specific fractions was analyzed. In the second step, the material from gel filtration (peak 1) was immediately dialyzed with distilled water at 4 °C and lyophilized. The active fraction (94 mg) was submitted to a DEAE column (1.0 × 19 cm) and equilibrated with 20 mM HEPES buffer (pH 7.5). Elution was carried out with a linear gradient of NaCl (0–0.3 M) at a flow rate of 11 mL/h. In the third step, peaks (C, E, and F) from the DEAE column containing hemorrhagic activity were joined, dialyzed with distilled water, concentrated (45 mg), and gel-filtered on a S-200 (1 × 100 cm) column. The column was developed with 20 mM HEPES buffer (pH 7.5), with 1 mM CaCl_2_ and 5 mM NaCl at a flow rate of 3.5 mL/h. The isolated peak (19 mg) of approximately 55-kDa by SDS-PAGE (12%), termed Bar-III, was dialyzed and lyophilized for further structural and biochemical characterization. An aliquot of this sample was applied on an RP-HPLC using a Vydac C18 (4.6 × 250 mm) column and equilibrated with 0.1% trifluoroacetic acid (TFA) in water (solution A). The enzyme was eluted using a linear gradient from solution A to 100% 0.1% TFA in acetonitrile (solution B) for 70 min at a flow rate of 1 mL/min. The elution was monitored by absorbance at 280 nm and analyzed by SDS-PAGE (12%).

### 5.4. Barnettlysin-III Purification

#### 5.4.1. Molecular Mass Determination by MALDI-TOF

MALDI-TOF mass spectra of Bar-III were performed using an Autoflex III MALDI-TOF-TOF mass spectrometer (Billerica, MA, USA) in a linear positive mode controlled by the proprietary COMPASSTM 1.2 software package. The Nd-YAG-laser power (355 nm) was manually adjusted for optimal signal appearance. A freeze-dried 0.5 µL solution of salt- and detergent-free protein in 30% ACN in 0.1% TFA was spotted on the ground steel target plate, mixed with 0.5 µL 10 mg/mL sinapinic acid in 50% ACN and 0.1% TFA, and left to dry at room temperature, next to standard protein mixtures for calibration.

The isolated proteinase showed an Mr of approx. 55 kDa estimated by reduced SDS-PAGE (12%) using the standard method of [87].

#### 5.4.2. Cloning and Sequencing of cDNA

Total RNA from *B. barnetti* venom was isolated, as described by [88]. cDNA synthesis was performed using the SuperScript First-Strand Synthesis System (Cat# 11904018, Invitrogen, Carlsbad, CA, USA). The cDNA-encoding PIII metalloproteinases were amplified via PCR with a Platinum™ SuperFi II DNA Polymerase (Cat# 12361010, Invitrogen), according to the instructions and using the primers proposed by [89]. The amplified products were inserted into the pCR2.1-TOPO vector using a TOPO TA Cloning Kit (Cat# K450002, Invitrogen) in accordance with the manufacturer’s guidelines, followed by transformation in One Shot Chemically competent TOP10 *E. coli* cells. Ampicillin-resistant transformants were screened by colony PCR with the SuperFi II Green PCR Master Mix (Cat#12369010, Invitrogen) using the M13 primers. The amplification products were sequenced on an ABI 3730 XL automated sequencer (Macrogen, Inc., Seoul, Republic of Korea) using M13 primers.

#### 5.4.3. In Silico Protein- and Sequence-Based Analysis

The obtained nucleotide sequences were examined against mRNA sequences in the GenBank database using the BLAST program (2007). The amino acid sequence of Bar-III was obtained from cDNA using the ORF finder program (2002) and was used for multiple alignments by the Clustal W 8.0 program (2004) with other homolog sequences in the UniProt database. The representation was generated using the ESPript server [90]. Domain organization and functional motifs were detected using the NCBI CD search tool [91]. The theoretical molecular mass and isoelectric point were predicted with the ExPASy ProtParam tool [92]. The three-dimensional model of Bar-III was established using the AlphaFold2 CoLab notebook v2.3.2. [93].

### 5.5. Enzyme Assays and Effect of Cations and Proteinase Inhibitors on Enzymatic Activity

Proteolytic activity was assayed with DMC, as described [94]. Purified Bar-III (1.5 µg) was incubated with CaCl_2_, MgCl_2_, ZnCl_2_, and CoCl_2_ (2 mM each) in 20 mM HEPES buffer (pH 7.4) for 15 min at 37 °C before the proteolysis of DMC was tested. Enzymatic activity was also assessed with various proteinase inhibitors: batimastat (BAT) and marimastat (MAR)—0.5 µM each; inhibitor of collagenase I (i-Coll-I) and inhibitor of matrix metalloproteinase-III (i-MMP-III) using 0.5 µM of each reagent; ethylenediaminetetracetic acid (EDTA) and phenylmethanesulfonil fluoride (PMSF) (1 mM each); and dythiotreitol (DTT) and chymostatin (Chy) (32 µM each). The residual enzymatic activity was evaluated with DMC.

### 5.6. Deglycosylation of Bar-III

To determine the content of the N-linked carbohydrate of Bar-III, 10 µg enzyme was dissolved in denaturing buffer (0.5% SDS, 1% β-mercaptoethanol, β-ME). The sample was denatured by boiling for 10 min and then incubated with glyco buffer, PN 40 (1%), and 2 units of recombinant PNGase F for 2 h at 37 °C. The reaction was stopped by boiling for 5 min. After the addition of a loading buffer, the reaction mixture, as well as the untreated control and deglycosylated proteinase, were submitted to SDS-PAGE. To preserve the activity of Bar-III, the protein was also deglycosylated for 1 h at 37 °C without the reduced and denaturing agents, as described in [14].

### 5.7. Enzymatic Activity on Fibrin(ogen), Fibronectin, Vitronectin, and Type IV and Type I Collagen

Human fibrinogen (Fg), fibrin (Fb), and fibronectin (FN) were incubated with Bar-III in a 1:100 (*w*/*w*) ratio at 37 °C in 50 mM Tris-HCl and 1 mM CaCl_2_ at pH 8.0. In the case of vitronectin (VT), incubation with proteinase was in a ratio of 1:150. The digestion of these protein substrates was also submitted to SDS-PAGE using 12% gels under reducing conditions to visualize the time sequence of degradation. Furthermore, an enzymatic effect was performed by incubating 1.5 µg Bar-III with 50 µL of each substrate for different time intervals at 37 °C.

### 5.8. Degradation of Matrigel

Matrigel matrix (Corning, Bedford, MA, USA) was applied to evaluate the effect of Bar-III on base membrane (BM) components. Matrigel is a solubilized BM-like composite from the Engelbreth–Holm–Swarm sarcoma surrogate of BM [95]. Its principal compounds are laminin, type IV collagen, nidogen, and heparan sulfate proteoglycans. Matrigel (50 µg) was incubated with Bar-III (1.5 µg) in 50 µL of 25 mM Tris-HCl, 150 mM NaCl and 2 mM CaCl_2_ (pH 8.0) at 37 °C in the presence or absence of 10 mM EDTA. Other experimental conditions have been described recently [24]. The Matrigel main components and their degradation products were transferred to a nitrocellulose membrane, submitted to immunoblotting, and detected with specific antibodies against collagen-IV (70R31139, Fitzgerald, 1:4000), laminin (NB300-144, Novus, 1:4000), nidogen (AF2570, RD Systems, 1:4000) (primary antibodies), and peroxidase-coupled protein G (1:1000 dilution) by chemiluminescence imaging systems (ChemiDoc).

### 5.9. Platelet Aggregation Assays

To analyze the effects of Bar-III on platelet aggregation, human platelet suspension was prepared from the whole blood of healthy volunteers who had used any medication in the past two weeks. Venous blood was collected in acid–citrate dextrose (ACD: 78 mM citrate acid, 117 mM sodium citrate, and 282 mM dextrose) [6.1, (*v*/*v*)] and immediately centrifuged at 600× *g* for 15 min, and the resulting supernatant platelet-rich plasma (PRP) was collected. Human washed platelets (WPs) were isolated, as described previously [96]. WPs were re-dispersed in Tyrode’s solution (pH 7.4) with 2 mM CaCl_2_ and 1 mM MgCl_2_ without PGE_1._ The number of platelets were adjusted to 2.5 × 10^5^/platelets/µL. Platelet aggregation was monitored by light transmission in an eight channel aggregometer (AggRam Helena Laboratories, Beaumont, TX, USA) with stirring at 600 rpm (37 °C).

### 5.10. Production of Anti-Bar-III Hyperimmune Sera

To produce anti-Bar-III sera, New Zealand rabbits weighing around 2 kg were used. The animals were housed in a controlled environment with a temperature range of 18 °C to 22 °C and provided with water and food. After collecting the pre-immune serum, the animals received subcutaneous injections with a final volume of 100 μL of an emulsion (1:1 *v*/*v*) prepared with complete Freund’s adjuvant and PBS (0.05 M phosphate buffer (pH 7.4) and 0.015 M NaCl) containing Bar-III, using variable and increasing masses of the toxin [97]. From the second immunization onward, emulsions were prepared with incomplete Freund’s adjuvant. All animals underwent four subsequent immunizations performed at 21-day intervals. Ten days after the last immunization, animal blood was drawn, and the serum was separated from the blood immediately after collection by centrifugation at 3000 RPM (5 min) and frozen at −20 °C. All protocols were approved by The Ethics Committee for Animal Use from the Biological Sciences Section of the Federal University of Paraná (CEUA/BIO—UFPR)—Statement No. 1308.

### 5.11. Cell Culture

Rabbit aorta endothelial cells (RAEC) [98] were grown in F12 medium (Gibco, Waltham, MA, USA) supplemented with 10% Fetal Bovine Serum (Gibco). Cells were cultured at 37 °C in a humidified environment with 5% CO_2_.

### 5.12. Cell Cytotoxicity Assays

For the cell viability test using trypan blue exclusion, RAECs (5 × 10^4^ cells) were grown in 12-well plates for 24 h. Then, the cells were incubated with Bar-III (5 μg/mL, 20 μg/mL, and 50 μg/mL) for 2 and 24 h. After, unadhered cells were collected and joined with cells released by treatment with 0.25% pancreatin/HBSS (Merck KGaA, Darmstadt, Germany).

Finally, the cell suspension was added to a 0.4% trypan blue solution (1:1 *v*/*v*), and viable (unstained) and non-viable cells (stained) were counted using a Microscope Leica MPS30 (Leica Microscopy System Ltd., Heerbrugg, Switzerland). The proportion of viable cells was calculated as displayed below:

$$viable\;cells\left(\%\right)=\frac{total\;number\;of\;viable\;cells\;per\;mL\;aliquot}{total\;cells\;per\;mL\;of\;aliquot}\times 100$$

Viability was also estimated using the CellTiter-Glo™ Kit (Madison, WI, USA), which determines the proportion of viable cells in a culture based on the ATP amount. For that, 2 × 10^4^ cells were seeded in 96-well plates for 24 h and then incubated with Bar-III (20 μg/mL, 50 μg/mL, and 100 μg/mL) for 2 and 24h. Controls used cell culture medium for background normalization and PBS instead of Bar-III. After these intervals, the CellTiter-Glo™ reagent was added (1:1 *v*/*v*), the plates were incubated for 10 min (at room temperature), and the absorbance (490 nm) was measured by a Tecan infinite^®^ M200 (Grödig, Áustria).

### 5.13. Cell Migration Scratch/Wound

A scratch/wound healing assay [99] was performed to examine cell migration. Cells were seeded (5 × 10^4^ cells/well) in a 96-well culture plate. After adherence, the medium was removed, and the medium without FBS was added. After 12 h, cells were treated with 10 μg/mL mitomycin C (Sigma) for 2 h. Then, the cell monolayer was scratched with a pipette tip. Immediately after scratching, the medium was discarded, and the cells were rinsed and incubated in a culture medium without FBS containing Bar-III in different amounts (10 μg/mL, 20 μg/mL, and 50 μg/mL). In addition, some wells were treated with Bar-III previously incubated with the metalloproteinase inhibitor marimastat™ (Sigma, St. Louis, MO, USA) (1.5 µg of marimastat/10 µg of Bar-III) or with PBS (the vehicle control) (1 h, 37 °C). Cells were then photographed after 0 h and 16 h. Relative migratory capacity was determined by calculating the percentage of the area between the two edges of the scratch using the Image J program v.1.8.0 [100].

### 5.14. Adhesion Assay

For the adhesion assay [101], before plating, RAECs in suspension (4 × 10^4^ cells) were treated (2 h) with Bar-III (10 μg/mL, 50 μg/mL, and 100 μg/mL) or Bar-III previously incubated with the metalloproteinase inhibitor marimastat^TM^ for 1 h at 37 °C (1.5 µg of marimastat^TM^/10 µg of Bar-III) or with vehicle (PBS). After that, cells were plated in microculture wells (96-well plates, TPP) pre-coated with fibronectin, vitronectin, or RGD peptides (10 μg/mL) and blocked with 1% BSA. Then, the cells were maintained at 37 °C for 2 h. After washing, the attached cells were fixed with methanol P.A. and stained with 0.8% crystal violet (Sigma, St. Louis, MO, USA) in 20% ethanol. Next, the plates were washed, and the dye from the cells was eluted with a solution of 50% ethanol in 0.1 M sodium citrate. Absorbance was measured at 490 nm using a MicroElisa Reader Automated Microplate Reader (ELX800-Bio Tek Instruments, Inc, St Clara, CA, USA). Control experiments were carried out with cells not treated with Bar-III. In this case, the cell adhesion was normalized to 100%. The negative control for cell adhesion was from microculture plates pre-coated with BSA, with adhesion normalized to 0%.

### 5.15. Flow Cytometric Analysis

At first, RAECs were released by treatment with 0.25% pancreatin/HBSS (Sigma), kept in an F12 medium, counted, and divided into microtubes (10^6^ cells/mL/tube). Cells were then added to the F12 medium containing 50 μg/mL of *Bar-III* for 2 h at 37 °C. Then, the cells were washed with PBS and incubated with 1% BSA/PBS for 20 min. Next, the cells were incubated with the Bar-III polyclonal antibodies 1:1000 in 1% BSA/PBS. The cells were then washed and incubated for 40 min with a solution of secondary rabbit anti-IgG antibodies conjugated with Alexa Fluor 488 diluted 1:50 (Donkey anti-Rabbit IgG secondary antibody Alexa Fluor 488, Invitrogen, Carlsbad, CA, USA). After another round of washing, cells were pelleted by centrifugation and fixed with 1% paraformaldehyde in PBS. A list mode file of 10,000 events was used to analyze each sample using a FACScalibur Becton Dickinson flow cytometer (Becton Dickinson Diagnostic Instrument Systems, Sparks, MD, USA). The presented data represent histograms of fluorescence frequency distribution. Per the antigen competition assay, the same immunofluorescence protocol was performed, but the Bar-III hyperimmune serum was previously incubated with 50 μg/mL of Bar-III for 1 h. The incubation mixture was then joined with endothelial cells, as described above.

### 5.16. Immunofluorescence and Fluorescence Cytochemistry Assays

The cells were seeded on glass coverslips, grown for 3 days, and then exposed to 50 µg/mL of Bar-III during 2 h at 37 °C. The medium of the control group contained equal amounts of vehicle (PBS). After washing with PBS, the cells were fixed with a solution of 2% paraformaldehyde for 30 min at 4 °C, incubated with 0.1 M glycine for 3 min, and washed again with PBS. After blockade with PBS with 1% BSA for 20 min, the cells were incubated with Bar-III antibodies (1:1000) for 1 h at room temperature and mouse monoclonal integrin alpha 5 antibody (Abcam, Cambridge, UK) 1:50 in 1% BSA/PBS, as suggested by the manufacturers. The cells were thoroughly washed and incubated with secondary antibodies conjugated with Alexa Fluor 488 (room temperature) for 40 min for α5 and Alexa Fluor 594 for Bar-III visualization. After washing, the slides were mounted with Fluoromount-G™ (Invitrogen) and observed using a confocal fluorescence microscope (Confocal Radiance 2100, Bio Rad Hercules, CA, USA) with a Nikon-Eclipse E800 with Plan-Apochromatic objectives (Sciences and Technologies Group Instruments Division, Melville, NY, USA).

### 5.17. Statistical Analysis

Statistical analysis of experiments was performed using ANOVA, with posthoc Dunn and Kruskal–Wallis tests. Values are presented as mean ± SD. Statistical significance was set as *p* < 0.05.

## Data Availability

The original contributions presented in the study are included in the article/Supplementary Materials, further inquiries can be directed to the corresponding author.

## Supplementary Materials

The following supporting information can be downloaded at: https://www.mdpi.com/article/10.3390/toxins16110486/s1, Figure S1: *B. barnetti* venom (1150 mg) was fractionated on Sephacryl S-200 column. Figure S2: The P1 fraction (94 mg) from first step was applied on DEAE Sepharose column. Figure S3. MALDI-TOF mass spectra of purified Bar-III. Figure S4: Immunodetection profile of anti-Bar-III polyclonal antibodies.
