# Supplementary material for: A Novel P-III Metalloproteinase from Bothrops barnetti Venom Degrades Extracellular Matrix Proteins, Inhibits Platelet Aggregation, and Disrupts Endothelial Cell Adhesion via α5β1 Integrin Receptors to Arginine–Glycine–Aspartic Acid (RGD)-Containing Molecules
Source: Toxins (Basel). 2024 Nov 9;16(11):486. doi: 10.3390/toxins16110486 (PMC11597958; doi:10.3390/toxins16110486)
Supplement: Supplementary file 1 [file toxins-16-00486-s001.zip › toxins-3242620-supplementary.pdf]

Supplementary materials

A Novel P-III Metalloproteinase from *Bothrops barnetti* Venom Degrades Extracellular Matrix Proteins, Inhibits Platelet Aggregation, and Disrupts Endothelial Cell Adhesion via $\alpha 5\beta 1$ Integrin Receptors to Arginine–glycine–aspartic acid (RGD)-Containing Molecules

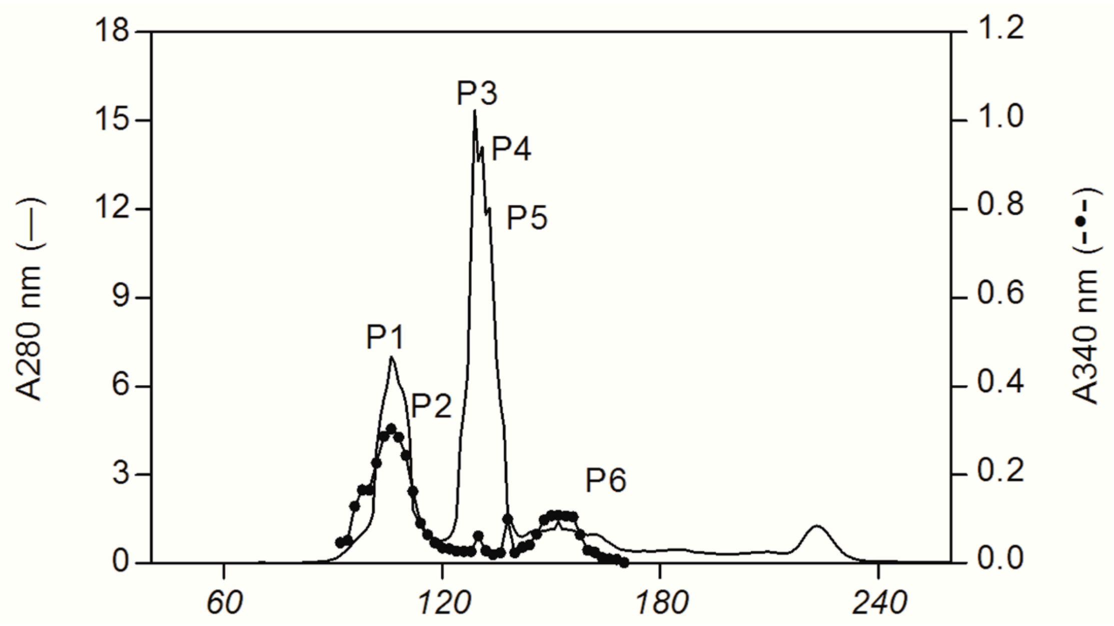

Figure S1. *B. barnetti* venom (1,150 mg) was fractionated on Sephacryl S-200 column. Two fractions (P1 and P6) showed proteolytic and hemorrhagic activities. The fraction P1 was pooled, dialyzed and lyophilized for the next step.

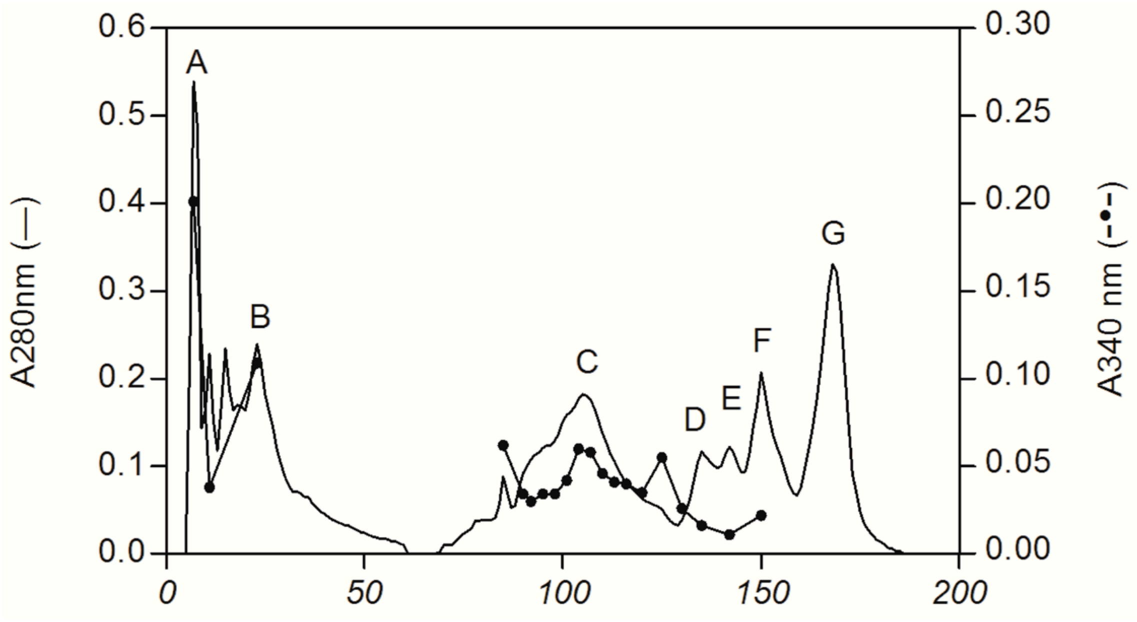

Figure S2. The P1 fraction (94 mg) from first step was applied on DEAE Sepharose column. The fractions D, E and F showed proteolytic and hemorrhagic activities. These fractions were pooled, dialyzed and lyophilized for the next step.

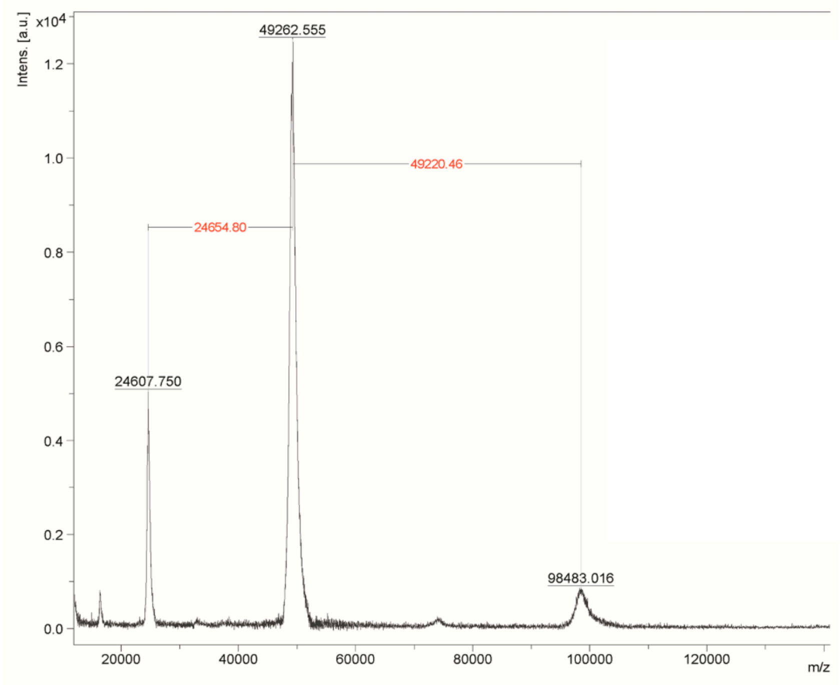

Figure S3. MALDI-TOF mass spectra of purified Bar-III. MS spectrum of Bar-III showed a 49,262.55 m/z and its doubly-charged (24,607.750 m/z).

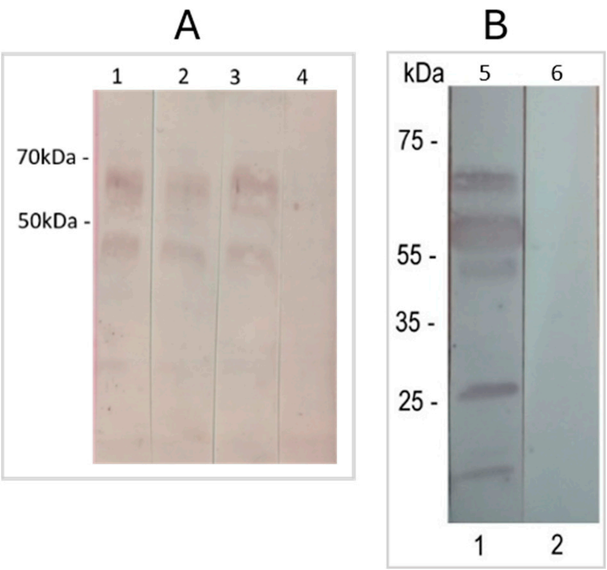

Figure S4. Immunodetection profile of anti-Bar-III polyclonal antibodies. (A) Detection of 2 µg of purified Bar-III by rabbit polyclonal antibodies at dilutions of (1) 1:3,000, (2) 1:5,000, and (3) 1:10,000. Two bands were observed, one likely a Bar-III degradation product (below 50 kDa standard). Column 4 shows antibody specificity control with pre-immune serum (1:3,000). (B) Detection of 2 µg of *Bothrops barnetti* venom by anti-Bar-III serum, showing a prominent 60 kDa band and others. Column 5 shows the antiserum reaction (1:3,000) and column 6 the pre-immune serum control (1:3,000).
